# Thermal and Mechanical Properties of Esterified Lignin in Various Polymer Blends

**DOI:** 10.3390/molecules26113219

**Published:** 2021-05-27

**Authors:** Alexander Orebom, Davide Di Francesco, Patrick Shakari, Joseph S. M. Samec, Clara Pierrou

**Affiliations:** 1 RenFuel K2B Materials AB, Rapsgatan 25, 754 50 Uppsala, Sweden; alexander.orebom@renfuel.se (A.O.); joseph.samec@su.se (J.S.M.S.); 2 Department of Organic Chemistry, Stockholm University, Svante Arrhenius Väg 16C, 106 91 Stockholm, Sweden; davide.di.francesco@su.se; 3 Ångström Laboratory, Department of Polymer Chemistry, Uppsala University, Lägerhyddsvägen 1, 751 21 Uppsala, Sweden; patrick.shakari@kemi.uu.se

**Keywords:** Kraft lignin, biopolymer, bioplastics

## Abstract

Lignin is an abundant polymeric renewable material and thus a promising candidate for incorporation in various commercial thermoplastic polymers. One challenge is to increase the dispersibility of amphiphilic lignin in lipophilic thermoplastic polymers We altered Kraft lignin using widely available and renewable fatty acids, such as oleic acid, yielding more than 8 kg of lignin ester as a light brown powder. SEC showed a molecular weight of 5.8 kDa with a PDI = 3.80, while the T_g_ of the lignin ester was concluded to 70 °C. Furthermore, the lignin ester was incorporated (20%) into PLA, HDPE, and PP to establish the thermal and mechanical behavior of the blends. DSC and rheological measurements suggest that the lignin ester blends consist of a phase-separated system. The results demonstrate how esterification of lignin allows dispersion in all the evaluated thermoplastic polymers maintaining, to a large extent, the tensile properties of the original material. The impact strength of HDPE and PLA blends show substantial loss upon the addition of the lignin ester. Reconverting the acetic acid side stream into acetic anhydride and reusing the catalyst, the presented methodology can be scaled up to produce a lignin-based substitute to fossil materials.

## 1. Introduction

Plastics are fundamental building blocks of numerous commercial material segments today, with a total production volume of about 359 million tons annually in 2018 [1]. Fossil plastics are strongly linked to environmental issues such as CO_2_ emissions and littering resulting in a widespread leakage of microplastics in the environment. With an increasing global population, the need for plastic components is expected to increase while the environmental concerns create a demand for technological development of renewable solutions [2]. Currently, the annual production capacity of bioplastics is less than 1% of the total plastic production volume, [3] which further emphasize the need for innovations to address the demand of renewable materials for fossil plastic substitution [4,5]. Lignin is a naturally occurring material and an abundant organic polymer as it is one of the main components in lignocellulosic biomass [6]. Kraft lignin is the main component in black liquor which is a waste product from the production of cellulose pulp. Although black liquor is integrated in a cyclic process of the paper mill where it is dewatered in evaporators and burned to produce energy for the mill and regeneration of process chemicals, the energy produced is excessive. Removal of lignin from this system has been shown to be economically favorable due to the possibility to increase the productivity of the pulp mill without investment in new CAPEX intensive recovery boilers [7,8,9].

One of the most economical ways to separate Kraft lignin from the stream is by two-step precipitation [10]. First, acidification of black liquor with carbon dioxide to pH 10, which leads to partial protonation of phenolic groups and promotes precipitation of lignin. After separation of the solid material, the precipitated lignin is fully protonated with sulfuric acid and washed with water to reduce the metal content. The final product is commercially available in bulk. Currently, three mills have implemented this technology only to increase the production of pulp [11]. Thus, precipitated Kraft lignin is already generated. Today, this lignin is burnt to a low value, and it would be worthwhile to find applications of higher value. Rigorous investigations of the Kraft lignin structure have been performed to reveal a more condensed structure compared to native lignin with very few β–O–4’ bonds and high content of aromatic and aliphatic alcohols [12,13].

Kraft lignin is a cheap and renewable material with high functionality and shows possible effects such as UV stabilization and thermostability. Kraft lignin currently has a limited commercial establishment but has been evaluated as a bio-based alternative for applications such as fuels, antioxidants, flame retardants, compatibilizers, and in combination with various plastics [6,14,15,16]. However, due to the scarce compatibility with lipophilic matrices, the direct blend of unmodified lignin with common plastics has not led to any commercial products, due to worse mechanical properties of the blend compared to the pure reference polymer in absence of any compatibilizers or grafting agent [17,18].

A common strategy to combine lignin with plastics has been a covalent modification to alter the physical properties of lignin material, reducing Tg and increasing lipophilicity. Esterification and alkylation of lignin using maleic anhydride and dichloroethane were attempted for polypropylene (PP) blends achieving miscibility; however, at 20% lignin ester loading the tensile strength decreased by >20% [19]. Promising results were obtained with acetate, propionate, and butyrate esters, [20] of which the latter was giving the most homogeneous blends with high-density polyethylene (HDPE). In addition, acid catalyzed lignin esterification was obtained by using a surfactant and oleic acid in water [21]. Ionic and covalent surface modification of lignin particles offers another strategy. Highly lipophilic agents such as cetyl trimethyl ammonium, dodecenyl succinic anhydride, and alkyl ketene dimer were used in an aqueous process to increase the compatibility of lignin with PP [22]. The thermal stability could be improved in polyvinyl chloride with various lignin esters at expense of mechanical properties [23]. The use of readily available fatty acids provides a cheap and renewable alternative to create a functionalized Kraft lignin with increased lipophilicity to improve the compatibility with common plastics such as PP, polyethylene (PE), and other hydrocarbon-based polymers. This can be achieved using an esterification procedure of the Kraft lignin with fatty acids [19,20,22,23,24,25]. The ease of ester bond formation and its relatively high thermal stability makes it an excellent choice of linker, simplifying the potential industrial-scale production and processability of plastic blends.

Our strategy to incorporate Kraft lignin into common plastics constitutes covalent modification using widely available and renewable fatty acids through a robust esterification methodology for FAEL production [26]. By using inexpensive chemicals and a simple process, the goal is to produce large quantities of lignin-based materials to meet the ever-growing need for fossil-free alternatives in plastic production.

## 2. Results and Discussion

### 2.1. Preparation of FAELs

**Figure 1:**
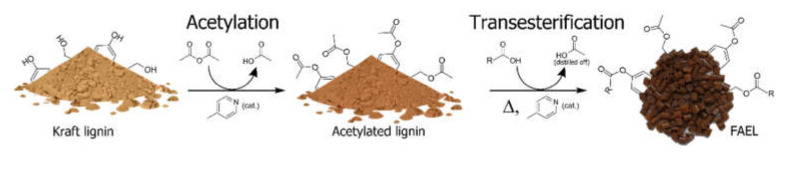
Schematic representation of the esterification mechanism.

As stated before, several approaches have been investigated to increase the lipophilicity of lignin to yield a material compatible with plastics. Among them, lignin esterification is one of the most promising for several reasons. One advantage is the possibility to utilize inexpensive and widely available bio-derived fatty acids further reducing the carbon footprint of the final product. However, previously reported procedures to yield FAELs involve the formation of acyl chlorides in situ [27,28], with the subsequent requirement of reagents and generation of a large amount of waste and therefore the requirement of additional chemicals to quench the acids generated during the process lowering the atom economy dramatically. This was circumvented by exploiting an organocatalyst (i.e., 4 methylpyridine) and an activator (acetic anhydride) recovering only acetic acid as a side product, where the organocatalyst was recycled. The esterification of Kraft lignin proceeds through the initial acetylation of hydroxyl groups of lignin. A further increase in temperature promotes a slow exchange of acetates to fatty acyl groups, where the resulting acetic acid can be recovered by distillation (Figure 1).

The acetic acid side stream can efficiently be re-converted to acetic anhydride by the well-established ketene method. By washing the product with kerosene, unreacted fatty acids can be also be recovered by distillation. Finally, utilizing acetic acid produced from bio-methanol carbonylation or fermentation of ethanol in the acetic anhydride synthesis leads to a negligible carbon footprint of the final product. Thus, the ester was produced on a pilot scale by mixing lignin, oleic acid, acetic anhydride, and 4-methylpyridine at 190 °C for 2 h. By the end of the reaction, 4 methylpyridine and acetic acid were recovered by distillation.

### 2.2. Analysis of FAELs

**Figure 2:**
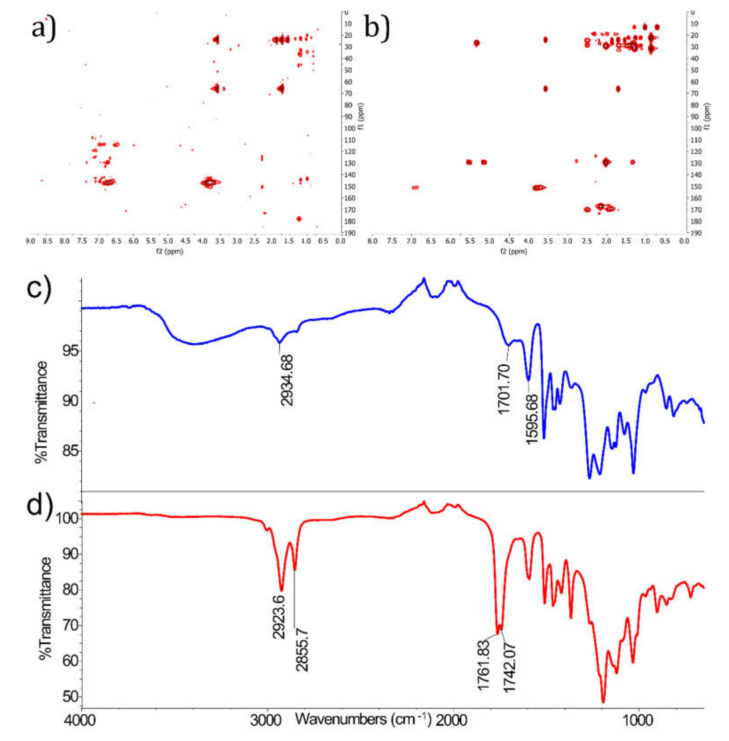
(**a**) HMBC spectrum of Kraft lignin, (**b**) HMBC spectrum of lignin FAEL, (**c**) FT-IR spectrum of Kraft lignin, and (**d**) FT-IR spectrum of FAEL.

To assure the success of the reaction several tests were performed on the esterified lignin. The melting point for FAEL was estimated to 105–140 °C. In addition, the FT-IR spectrum of FAEL showed two peaks in the area corresponding to the stretching mode of carbonyl groups of esters bounded to alkyl and aromatic substituents, 1742 and 1761 cm^−1^ respectively, [29] while the frequencies of free acetic and oleic acid associated to the same vibrational mode correspond to 1707 cm^−1^ (Figure 2, Figure S6). ^1^H NMR and HMBC studies confirmed the success of the esterification reaction: by comparing the spectra of FAEL with the initial substrate, i.e., Kraft lignin, it was possible to distinguish peaks corresponding to the ester moieties, e.g., alpha protons of fatty esters corresponding to the area comprised in 165–175 ppm, 1.75–2.55 ppm on the HMBC spectra (Figure 2, Figures S7–S10).

### 2.3. Thermal Properties

**Figure 3:**
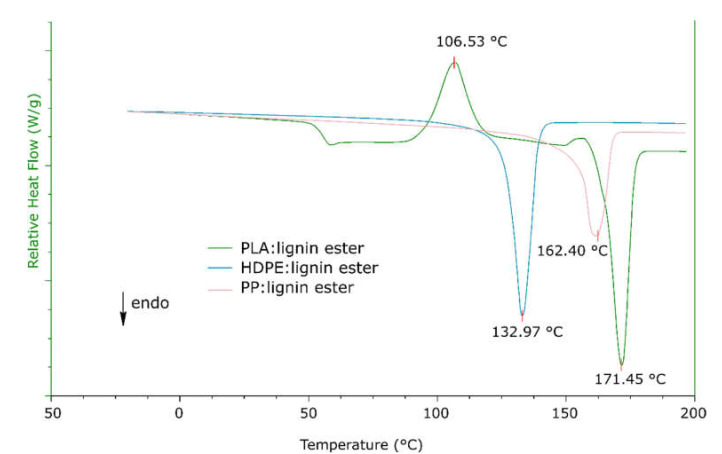
DSC measurement of the second heating cycle for composites of FAEL with PLA, HDPE, and PP respectively.

The DSC curve for pure FAEL shows a T_g_ of 70 °C and using TGA, the decomposition temperature was concluded to 275 °C. No signs of weight loss were found at 100 °C, an indication that the FAEL samples contain no or very little water. The complete DSC and TGA curves for the pure FAEL can be found in Figures S1 and S2. Figure 3 present the DSC curve throughout the second heat cycle for the various polymer blends. In Figure 2, the T_g_ for the PLA blend is indicated at around 60 °C from the second heating cycle. The DSC measurement further show exothermic reactions for crystallization at 106 °C and 160 °C, in consistency with literature [30,31]. The shift of exothermic peaks upon cooling of the samples indicates a change in the molecular structure of the samples after heating. Noticeably, no phase separation can be observed for the PP and HDPE blends using DSC measurements (Figure 2). The PP and HDPE blends show an exothermic reaction for crystallization upon cooling at 116 °C and 120 °C respectively which can be seen in the full DSC cycle (Figures S4 and S5), a behavior not seen for PLA, which could be due to rapid cooling.

### 2.4. Blending of FAELs with Polymeric Matrices

**Figure 4:**
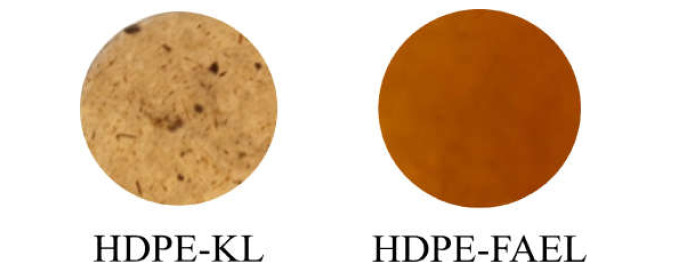
Comparison of round samples thin (thickness 1 mm, diameter 5 cm, loading 6 wt %) of unmodified Kraft lignin (**left**) and lignin ester (**right**) dispersed in polyethylene.

In order to measure the contribution of the sole esterification to the mechanical properties of the final blend, the compatibility between unmodified Kraft lignin and HDPE was initially studied. However, under our conditions, the scarce miscibility of Kraft lignin into polyolefins did not allow the production of a blend with a level of homogeneity suitable for mechanical testing. Therefore, unmodified Kraft lignin was not included in our study. Nevertheless, FAELS gave promising results (Figure 4).

**Figure 5:**
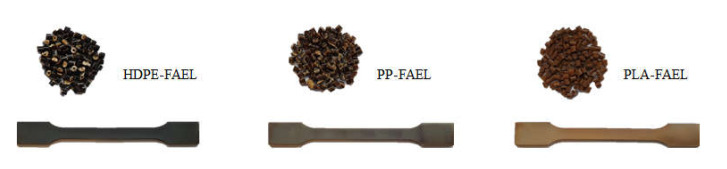
Picture of prepared granules of the FAEL polymer blends together with dumbbell samples for tensile testing. Lignin ester polymer blends in HDPE matrix (**left**), PP matrix (**center**), and PLA matrix (**right**).

Figure 5 illustrates the prepared granules and dumbbell samples of the polymer blends for tensile testing. The PLA blend shows a copper tint in color, which is assumed to be due to phase separation against the metal surface of the mold during injection molding. The remaining blends show a dark brown tint. The difference in color between the polymer blends is suggested to be due to the different morphologies of each matrix.

### 2.5. Rheological Properties

**Figure 6:**
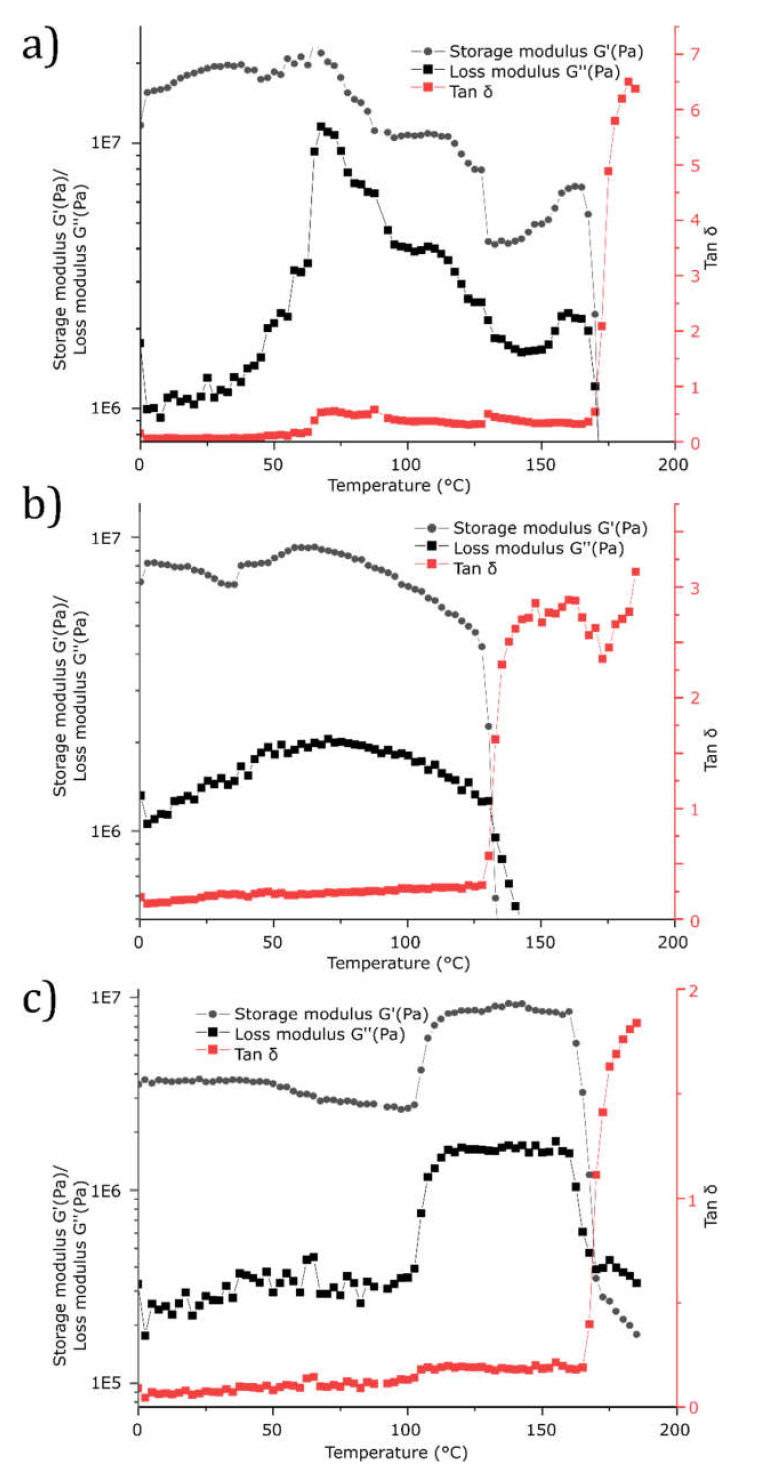
Temperature ramp of (**a**) PLA-FAEL, (**b**) HDPE-FAEL, and (**c**) PP-FAEL.

Using rheology as a tool for characterization, the various blends were studied using temperature ramps up to 185 °C to cover all phase changes as confirmed using the DSC measurements (Figure 6) to investigate the mechanical behavior and blend morphology as a function of time upon heating. As seen in Figure 6a, the PLA blend shows a substantial shift in loss modulus upon reaching 49 °C for which the DSC measurements of the same sample suggest an occurrence of Tg. Temperature range 75–96 °C show a steep decrease in G’, a clear sign of the crystallization phase in the blend which further is confirmed by the DSC measurement. A recrystallization phase is spotted at 133 °C before the material completely melt at 173 °C. Commonly, one issue using PLA is its low-rate crystallization and even though the comparison with pure PLA to establish any improvements in crystallization rate is included in this study, it would be a factor of interest for further research. Figure 6b suggests that the HDPE blend show a relatively stable structure with small molecular rearrangements up to 65 °C, and a complete melt of the blend at 135 °C. The PP blend morphology is relatively stable up to 100 °C, as seen in Figure 6c before the stiffness increase with a large margin. By comparing Figure 3 at temperature range 100–130 °C in the cooling cycle and Figure 6c, a sufficiently slow temperature ramp is applied to enable crystallization rather than melting.

### 2.6. Mechanical Properties

Relative mechanical properties evaluated according to ISO 527-2 and ISO 179 and calculated according to the mechanical properties of lignin blends divided by mechanical properties of the reference polymer. In Table 1, the relative mechanical properties for the polymer blends are presented. The numerical values for the mechanical properties of the blends can be found in Tables S1 and S2. The results show a decrease in impact strength for all blends, for which the HDPE blend show substantial performance loss upon the addition of FAEL in the matrix. However, the PP blend shows just a slight performance loss with regards to the impact strength upon the addition of FAEL. Throughout the results for the elongation at break, the PLA blend shows promising performance since almost no tensile properties are affected. These results indicate adequate interactions between the matrix polymer and FAEL in the blend. Tensile properties for the PP blend show indications of a slightly more brittle material, whereas HDPE in this case has slightly less affected properties in comparison to the reference.

## 3. Materials and Methods

### 3.1. Materials

Commercial chemicals were obtained from Sigma-Aldrich and used without further purification. The Kraft lignin obtained from the LignoBoost Demo plant from Bäckhammar, Sweden was ground on a Retsch GM300 mixer and dried at 60 °C overnight. Technical oleic acid (90%) was purchased from Sigma-Aldrich. Large scale reactions were performed in a Büchiglasuster 60 L CR60-C reactor lined with PTFE and heated by Huber unistat T330. Materials used to prepare the polymer blends were commercial-grade PP, HDPE, and polylactic acid (PLA). PP (HF136MO) and HDPE (MG9647S) was supplied by Borealis AH and PLA (Luminy L105) was supplied by Total Corbrion PLA 

### 3.2. Analysis

Size exclusion chromatography (SEC) was performed on a YL 9110 HPLC-GPC system (YL Instrument Co. Ltd., Dongan-gu, Anyang-si, Kyounggi-do, 431836, The Republic of Korea) with three Styragel columns (HR 0.5, HR 1, HR 3 7.8 × 300 mm each) connected in series (flow rate: 1 mL min^−1^; injection volume: 50 µL; solvent: THF), with a UV detector (280 nm) and autosampler. The system was calibrated using ReadyCal-Kit Polystyrene. (MP 266, 682, 1250, 2280, 3470, 4920, 9130, 15,700, 21,500, 28,000, 44,200, 66,000 Da). Thermogravimetric analysis (TGA) was performed on a Mettler TGA/DSC1 system using a temperature program from 50 °C to 600 °C (heating rate: 10 °C min^−1^) in a nitrogen atmosphere (flow rate: 80 mL min^−1^). The samples were held at a constant temperature in 5 min, followed by the airflow of 80 mL min^−1^ at 600 °C for 10 min. Differential scanning calorimetry (DSC) was performed on a DSC Q2000 system using a heat/cool/heat program from −25 °C to 200 °C (heating rate: 10 °C min^−1^, cooling rate: 5 °C min^−1^). Tensile tests were performed using a MTS20/M system according to ISO 527-2 (1 mm min^−1^/50 mm min^−1^), and impact strength tests were performed using an Instron CEAST 9050 instrument according to ISO 179 type A using a 0.5 J hammer with sample sizes of 3.94 × 8 × 8 mm. Rheological measurements were performed using a Discovery Hybrid Rheometer 2 with 8 mm stainless steel geometry heads (DHR-2, TA Instruments, Sollentuna, Sweden) using a temperature ramp function (strain: 0.01–0.02%, temperature range: 0–185 °C, heating rate: 2.5 °C/5 s) using samples in circular discs with 10 mm in diameter. NMR spectra were recorded on a Bruker 400MHz spectrometer. All spectra were processed using MestreNova from Mestrelab Research S.L. The chemical shifts are shown in parts per million (δ) relative to d8-THF CHD (3,4) (1.72 ppm for ^1^H and 25.31 ppm for ^13^C) and d8-THF CHD (2,5) (3.58 ppm for 1H and 67.21 ppm for ^13^C) as the internal standard. IR spectra were recorded on a Perkin-Elmer Spectrum-100 FT-IR spectrometer with an ATR accessory. Neat samples were analyzed by placing them directly onto the ATR crystal.

### 3.3. Synthetic Procedures

#### 3.3.1. Preparation of Fatty Acid Ester of Lignin (FAEL)

In a pilot reactor equipped with a mechanical stirrer and a downward distillation column were added the oleic acid (3.60 kg) and 4-methylpyridine (6.00 L). The temperature of the reactor heating mantle was raised to 120 °C and Kraft lignin (6.00 kg) was added in portions. The mixture was stirred for 10 min and acetic anhydride (4.50 L) was added over 6 min, temperature was raised to 190 °C, and kept there for 1 h. The pressure was lowered gradually to 20 mbar over 1 h and the acetic acid and catalyst were distilled off. Kerosene (6 L, b.p. 175–245 °C) was added and distillation under vacuum was continued over 1 h. On cooling, 10.53 kg of the product was collected. The material was ground to a fine powder and washed with petroleum ether (15 L to 4 kg) at 40 °C for 2 h, filtered and washed with warm (40 °C) petroleum ether (6 L). The solids were stirred with petroleum ether (10 L) overnight, filtered, and washed with petroleum ether (5 L). The filter cake was dried in a stream of air to remove volatile components. The workup of the whole batch afforded 8.53 kg material as a light brown powder with a molecular weight (Mw) of 5.8 kDa according to SEC with a PDI of 3.80 (Figure S11). By washing the product with kerosene, unreacted fatty acids were recovered by distillation for later reuse.

#### 3.3.2. Blend Preparation

Polymer blends of FAEL in PP, HDPE, and PLA were prepared using a Coperion twin-screw compounder to mix blends with a 20% FAEL to plastic proportion and pure samples of PP, HDPE, and PLA as reference material at 180 °C. The obtained extrudates were cut using a granulator into pieces followed by injection molding of the pellets into dumbbell-shaped samples using an Engel injection molder at 185 °C with reference to ISO 527-2 specifications. The dumbbell samples were used to determine the tensile properties of each FAEL sample compared to the reference, respectively.

## 4. Conclusions

Using lignin esterification with oleic acid, this study suggests a route of modification to enhance the dispersibility of lignin with various commercial polymers. FAELs as a component in PLA, HDPE, and PP blends exhibit uncomplicated processability using compounding and injection molding as well as promising mechanical and thermal properties. The tensile properties of the blends are to some extent maintained in the blends compared to the reference polymers, an indication of interactions between the FAEL and polymer matrix, respectively. Concerning the impact strength of the various blends, the results highlight the need for further research of the molecular structure using microscopical methods to derive the decrease to a specific cause. By utilization of inexpensive and readily available chemicals, the method of functionalization shows the potential of industrial establishment and large-scale production of sustainable lignin-based materials. With the potential of re-conversion of the acetic acid side stream into acetic anhydride and reuse of catalyst, the presented method of FAEL production may be established at a large scale as a fully renewable substitute to fossil materials. These lignin esters constitute a promising candidate to increase the availability of bio-based alternatives, which especially relevant considering the urgent global need for sustainable materials.

## Figures and Tables

**Table 1:** Values of relative mechanical properties for FAEL blends with PP, HDPE, and PLA.

	PP-FAEL/PP	HDPE-FAEL/HDPE	PLA-FAEL/PLA ^1^
Stress at yield	0.85	0.96	0.99
Strain at yield	0.50	0.75	0.88
Young’s modulus	1.09	1.05	0.93
Impact strength	0.93	0.39	0.50

^1^ [32].

## Data Availability

Data is contained within the article and supplementary materials.

## Supplementary Materials

The following are available online, Figures S1–S5: detailed TGA and DSC spectra of blends; Figure S6: FT-IR spectra of lignin and esterified lignin; Figures S7–S10 NMR spectra of lignin and esterified lignin; Figure S11: GPC chromatogram of lignin ester and kraft lignin; Tables S1–S2: Numerical values for mechanical properties of the various blends and references.

## Sample Availability

Not available.
