# Supplementary material for: Thermal and Mechanical Properties of Esterified Lignin in Various Polymer Blends
Source: Molecules. 2021 May 27;26(11):3219. doi: 10.3390/molecules26113219 (PMC8198513; doi:10.3390/molecules26113219)
Supplement: Supplementary file 1 [file molecules-26-03219-s001.zip › molecules-1174636-supplementary.pdf]

Electronic Supporting Information

# Thermal and mechanical properties of esterified lignin in various polymer blends

Alexander Orebom[a], Davide Di Francesco[b], Patrick Shakari[c], Joseph Samec[a,b] and Clara Pierrou*,[a]

[a] RenFuel K2B Materials AB, Rapsgatan 25, 75450 Uppsala (Sweden)

[b] Department of Organic Chemistry, Arrhenius Laboratory, Stockholm University, Svante Arrhenius väg 16C, 106 91 Stockholm (Sweden)

[c] Department of Polymer Chemistry, Ångström laboratory, Uppsala University, Lägerhyddsvägen 1, 751 21 Uppsala (Sweden)

**Thermogravimetric analysis**

Thermogravimetric analysis (TGA) was performed on a Mettler TGA/DSC1 system using a temperature programme from 50°C to 600°C (heating rate: 10°C min$^{-1}$) in nitrogen atmosphere (flow rate: 80 mL min$^{-1}$). The samples were held at constant temperature in 5 min, followed by an air flow of 80 mL min-1 at 600°C for 10 minutes. Sample size 5 mg.

^exo

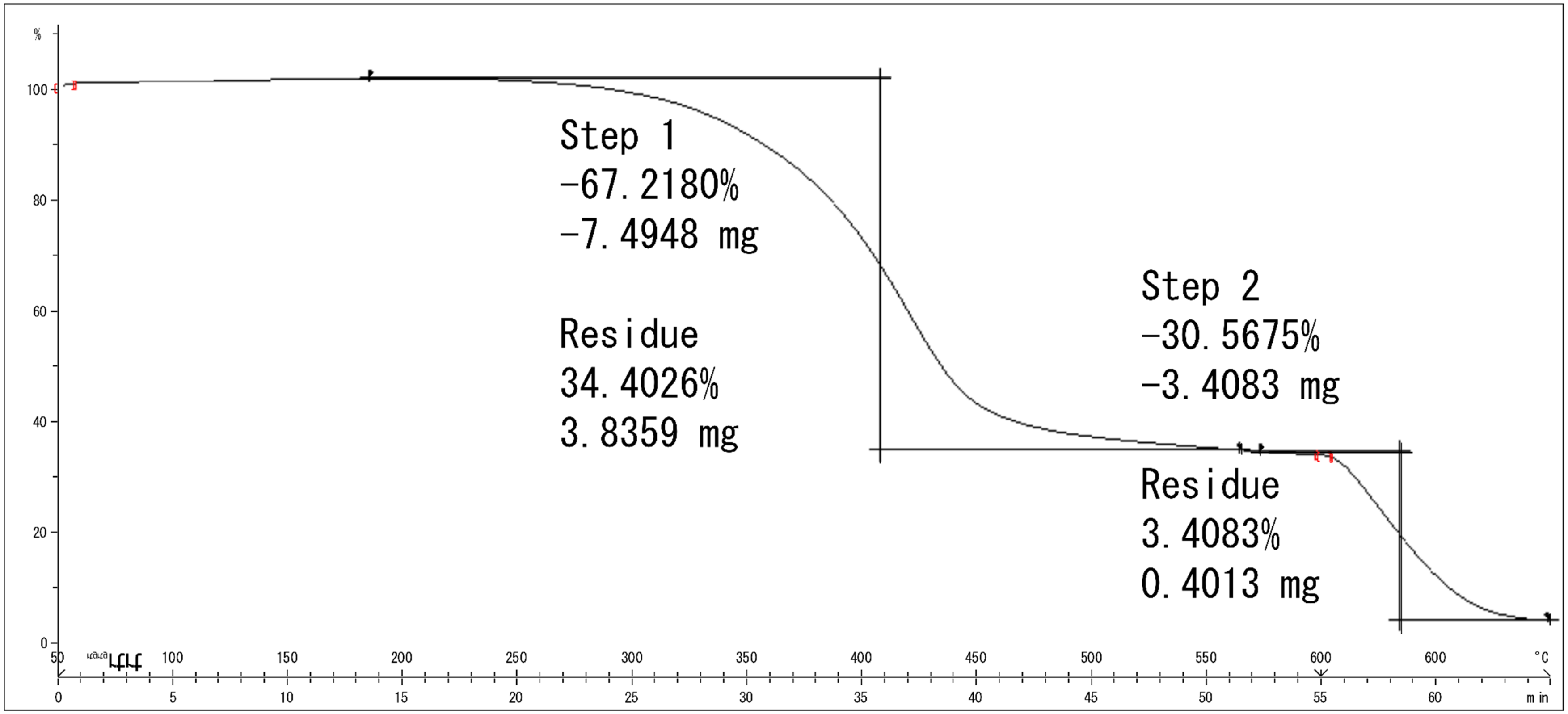


*Figure S1 TGA curve for pure lignin ester.*

## Differential scanning calorimetry

Differential scanning calorimetry (DSC) for the blends was performed on a DSC Q2000 system using a heat/cool/heat programme from -25°C to 200°C (heating rate: 10°C min -1, cooling rate: 5°C min -1). DSC for pure lignin ester was performed on a Mettler DSC1 system using a heating programme from 25°C to 175°C (heating rate: 10°C min -1) in nitrogen atmosphere (flow rate: 80 mL min $^{-1}$). Sample size 5 mg.

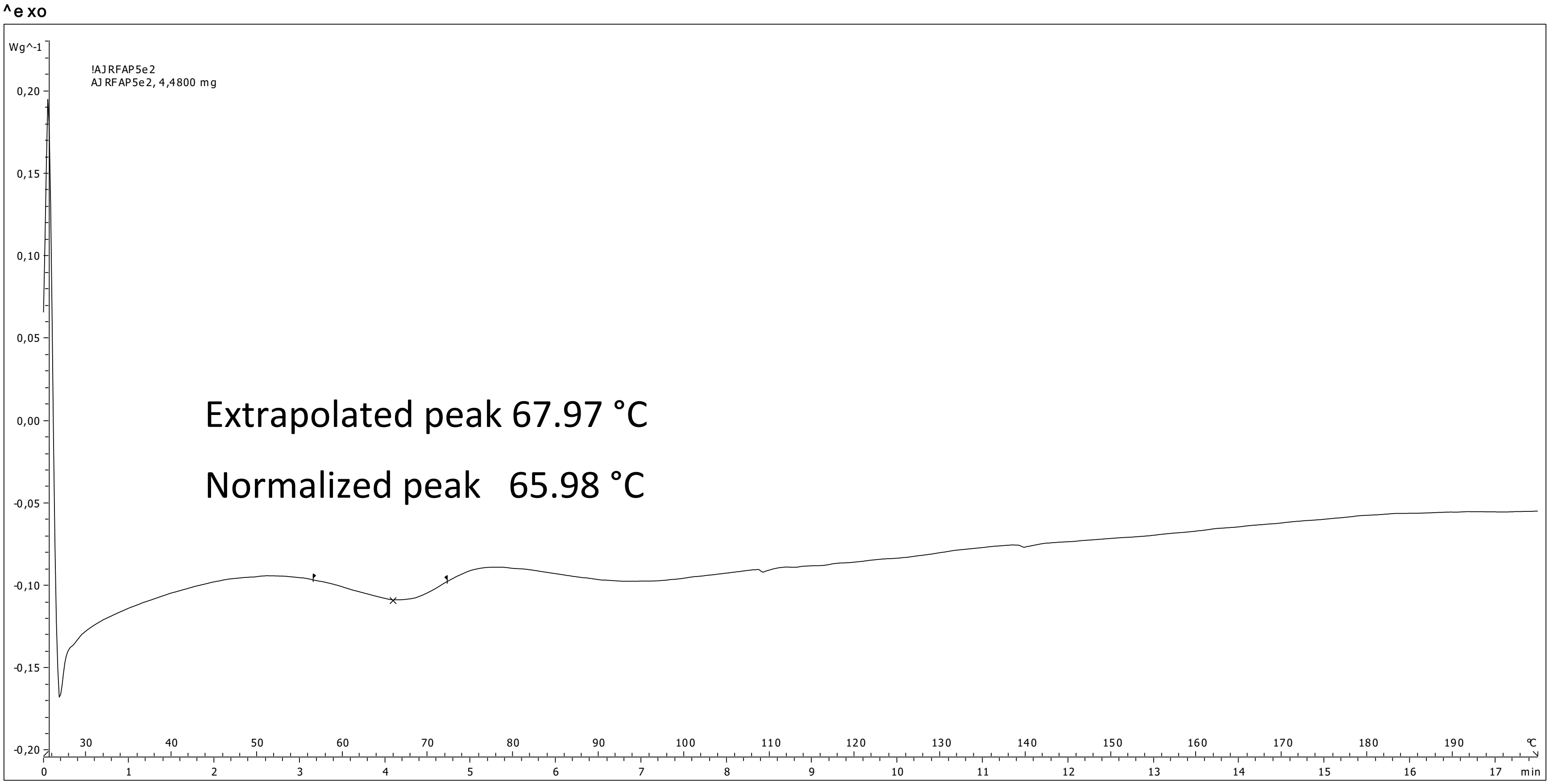


*Figure S2 DSC curve for pure lignin ester.*

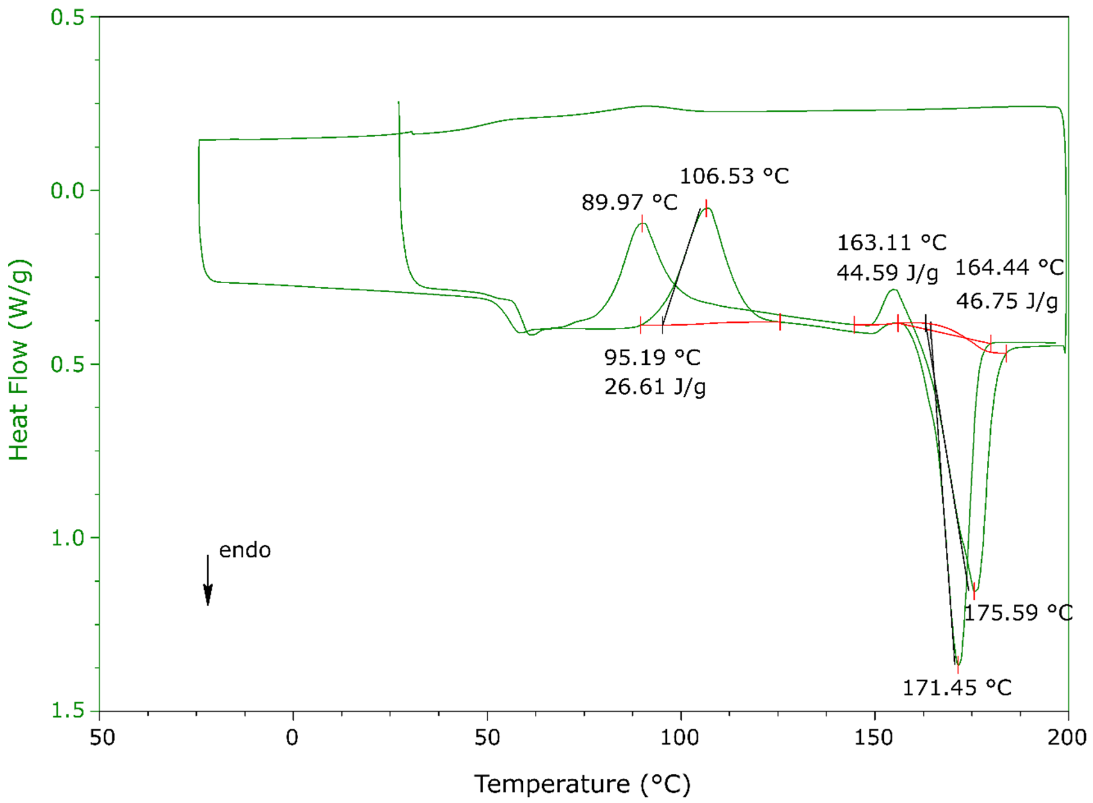


*Figure S3 DSC curve for PLA – lignin ester using heat/cool/heat programme from -25°C to 200°C (heating rate: 10°C min$^{-1}$, cooling rate: 5°C min$^{-1}$).*

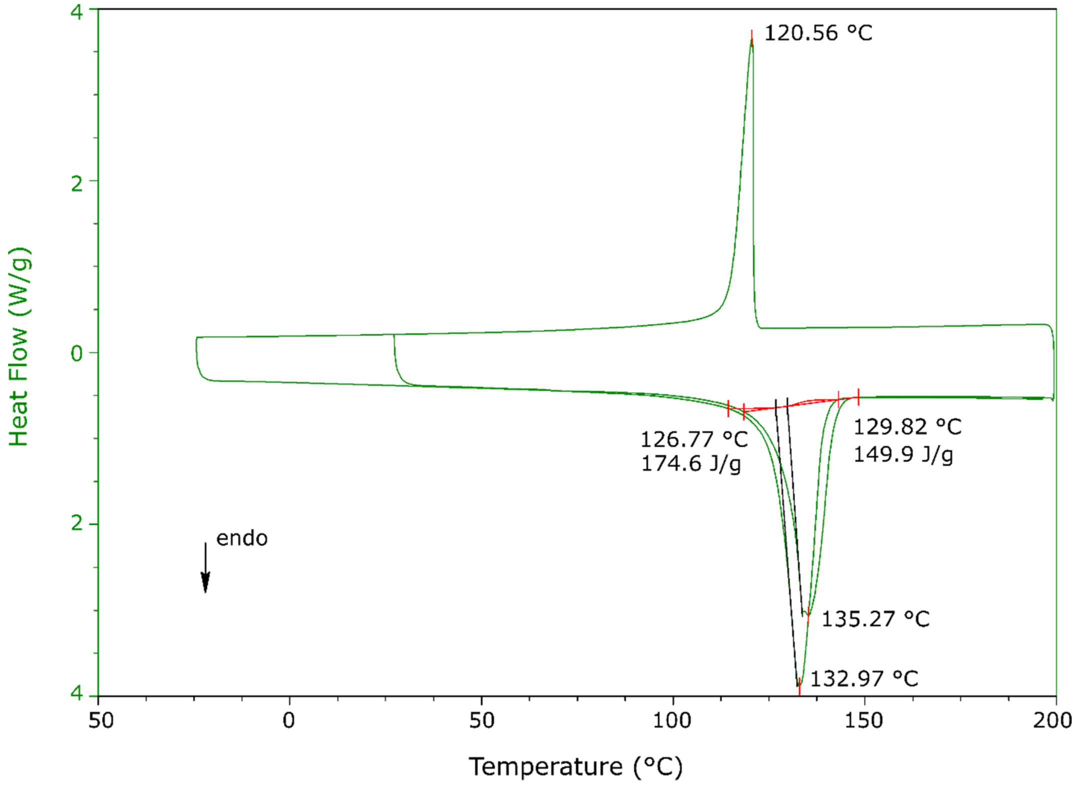


*Figure S4 DSC curve for HDPE – lignin ester using heat/cool/heat programme from -25°C to 200°C (heating rate: 10°C min$^{-1}$, cooling rate: 5°C min$^{-1}$)*

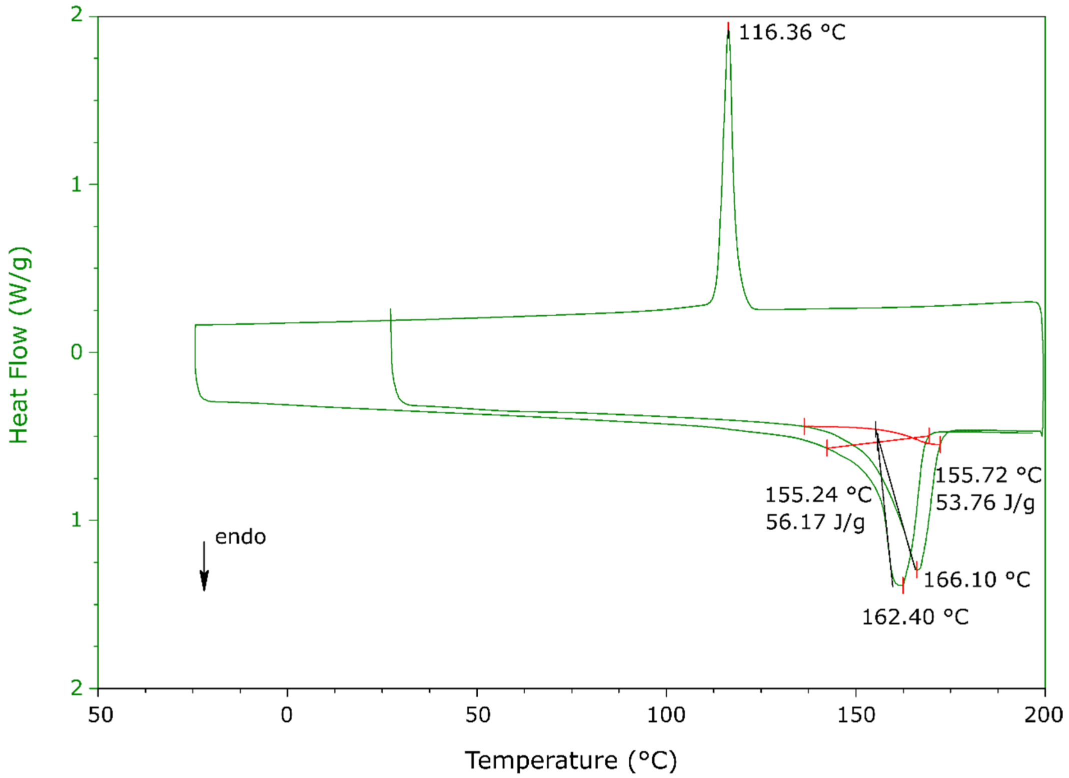


*Figure S5 DSC curve for PP – lignin ester using heat/cool/heat programme from -25°C to 200°C (heating rate: 10°C min$^{-1}$, cooling rate: 5°C min$^{-1}$).*

**FT-IR**

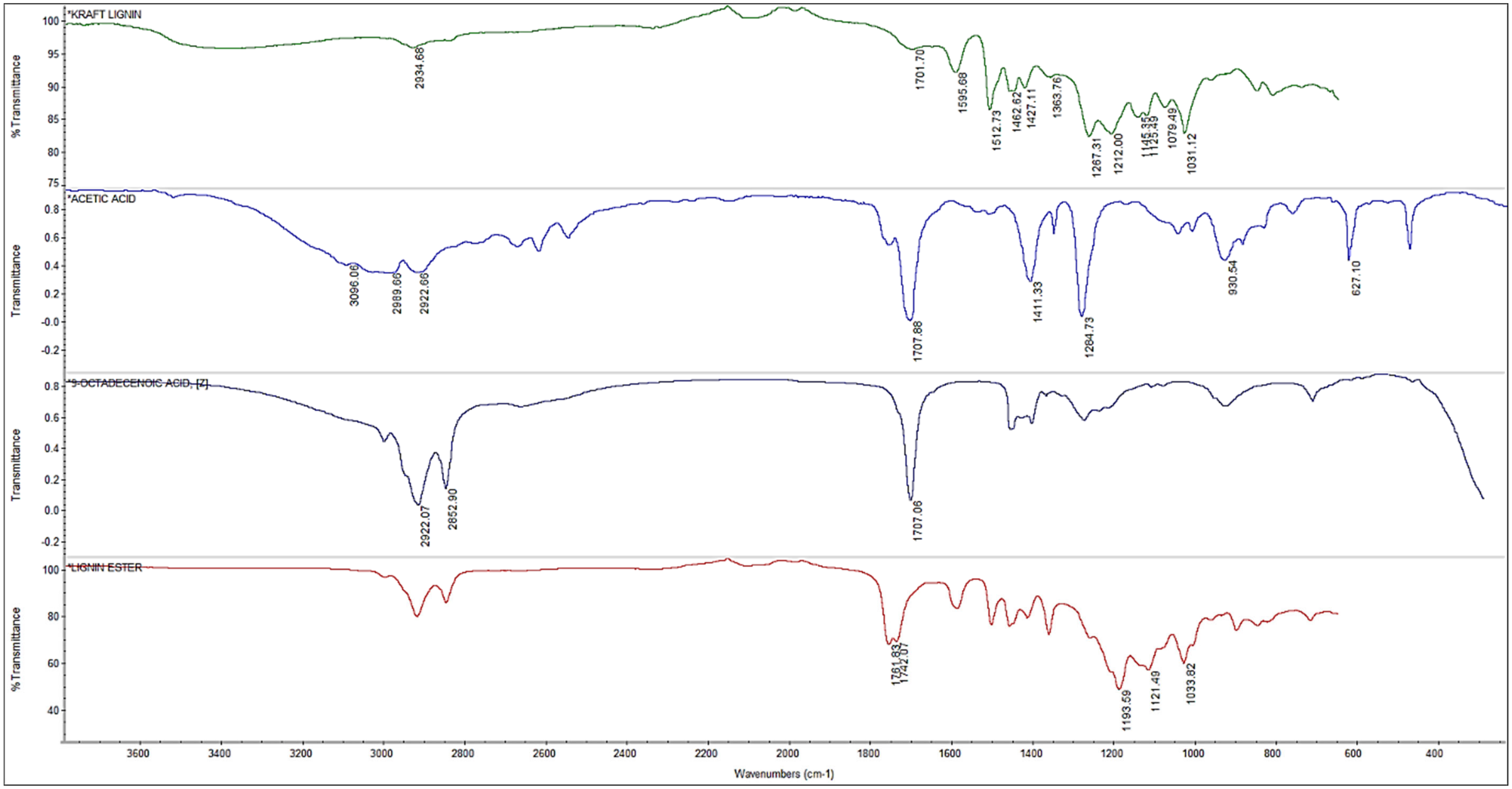


*Figure S6 FT-IR spectra of (from top to bottom) kraft lignin, acetic acid, oleic acid, and lignin ester.*

## NMR

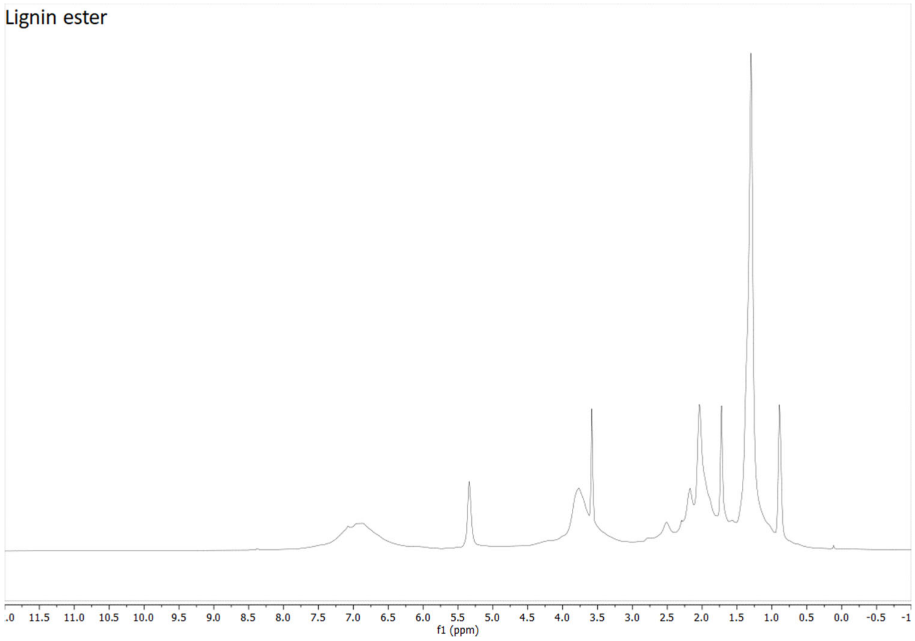


*Figure S7 $^1$H NMR spectrum of lignin ester in d8-THF.*

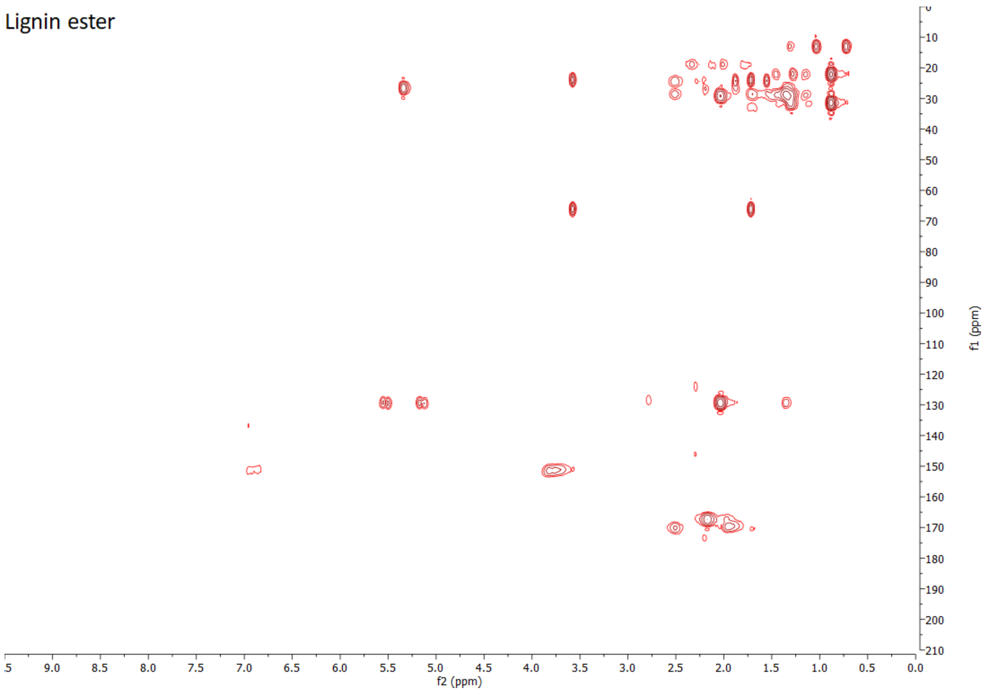


*Figure S 8 HMBC spectrum of lignin ester in d8-THF.*

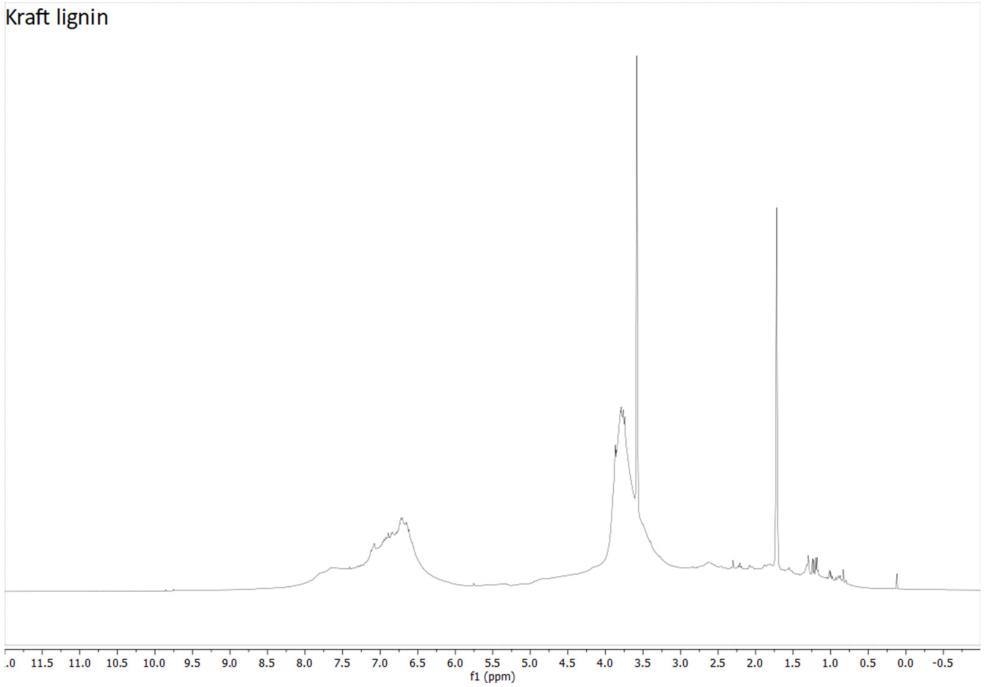


*Figure S9 $^{1}$H NMR spectrum of Kraft lignin in d8-THF.*

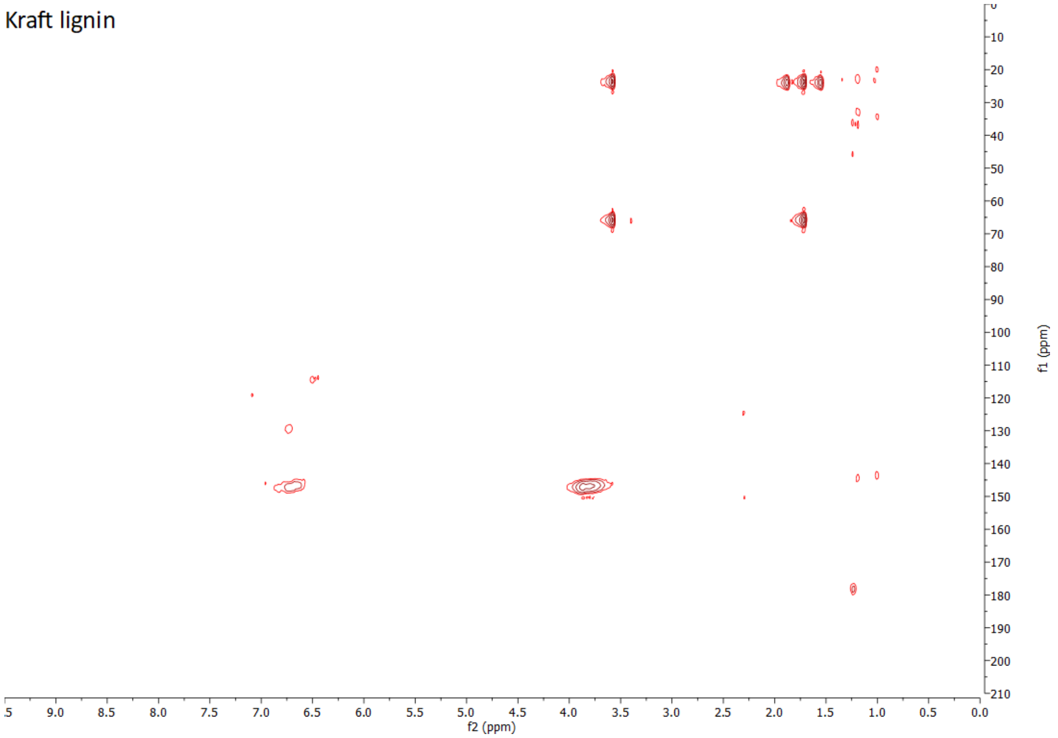


*Figure S10 HMBC spectrum of Kraft lignin in d8-THF.*

## GPC

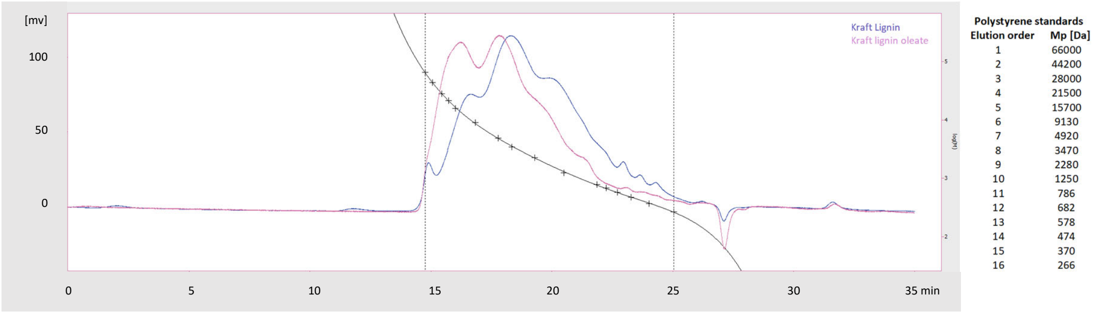


*Figure S11 GPC chromatogram of lignin ester (pink) and kraft lignin (blue).*

## Mechanical properties

Polymer blends of lignin esters in PP, HDPE and PLA were prepared using a Coperion twin screw compounder to mix blends with a 20% lignin ester to plastic proportion and pure samples of PP, HDPE and PLA as a reference material at 180°C. The obtained extrudates were cut using a granulator into pieces followed by injection moulding of the pellets into dumbbell shaped samples using an Engel injection moulder at 185°C with reference to ISO 527-2 specifications. The dumbbell samples were used to determine the tensile properties of each lignin ester sample compared to the reference, respectively. Tensile tests were performed using a MTS20/M system according to ISO 527-2 (1 mm $min^{-1}$ /50 mm $min^{-1}$), and impact strength tests were performed using an Instron CEAST 9050 instrument according to ISO 179 type A using a 0.5 J hammer with sample sizes of 3.94x8x8 mm.

*Table S1 Numerical values for mechanical properties of the various blends.*

| | | | **HDPE – LE** | | **PP – LE** | | **PLA – LE** | |
|---|---|---|---|---|---|---|---|---|
| **Tensile properties** | Method | Unit | Value | δ | Value | δ | Value | δ |
| Young's modulus | ISO 527-2 | MPa | 1260 | 18 | 1637 | 35 | 3248 | 31 |
| Stress at yield | ISO 527-2 | MPa | 26.3 | 0.2 | 29.4 | 0.2 | 59.4 | 0.2 |
| Strain at yield | ISO 527-2 | % | 6.3 | 0.2 | 3.9 | 0.1 | 2.1 | 0.1 |
| **Impact resistance (+23 °C)** | | | | | | | | |
| Charpy. notched. | ISO 179 | $kJ/m^2$ | 2.7 | 0.7 | 2.8 | 0.5 | 2.1 | 0.6 |
| Charpy. unnotched. | ISO 179 | $kJ/m^2$ | 25.6 | 2.8 | 17.5 | 1.1 | 14.8 | 1.6 |

*Value intended as mean value and δ as standard deviation.*

*Table S2 Numerical values for mechanical properties of the reference polymers.*

| **Tensile properties** | Unit | **HDPE** | **PP** | **PLA** |
|---|---|---|---|---|
| Young's modulus | MPa | 1200 | 1500 | 3500 |
| Stress at yield | MPa | 27 | 34 | 60[1] |
| Strain at yield | % | 8 | 9 | 2.4[1] |
| **Impact resistance (+23 °C)** | | | | |

| | | | | |
|---|---|---|---|---|
| Charpy. notched. | $kJ/m^2$ | 7 | 3 | 5 |

## References


1 G. W. de Kort. L. H. C. Bouvrie. S. Rastogi and C. H. R. M. Wilsens. *ACS Sustain. Chem. Eng.*. 2020. **8**. 624–631.

2 Acetic acid. https://webbook.nist.gov/cgi/cbook.cgi?ID=C64197&Type=IR-SPEC&Index=2#IR-SPEC. (accessed 2 November 2020).

3 Oleic Acid. https://webbook.nist.gov/cgi/cbook.cgi?ID=C112801&Units=SI. (accessed 2 November 2020).
